# Epidemiological Characteristics of Postoperative Sepsis

**DOI:** 10.1515/med-2019-0110

**Published:** 2019-12-31

**Authors:** Po-Yi Chen, Ci-Wen Luo, Mu-Hsing Chen, Ming-Ling Yang, Yu-Hsiang Kuan

**Affiliations:** 1 Department of Pharmacology, School of Medicine, Chung Shan Medical University; Department of Pharmacy, Chung Shan Medical University Hospital, No.110, Sec. 1, Jianguo N. Rd, Taichung, Taiwan, Republic of China; 2 Department of Optometry, Chung Hwa University of Medical Technology, Tainan, Taiwan; 3 Department of Optometry, DAYEH University of Medical Technology, Taichung, Taiwan; 4 Department of Anatomy, School of Medicine, Chung Shan Medical University, Taichung, Taiwan

**Keywords:** Incidence, Epidemiological characteristics, Risk of postoperative sepsis, NHIRD

## Abstract

**Background:**

Postoperative sepsis is a major type of sepsis. Sociodemographic characteristics, incidence trends, surgical procedures, comorbidities, and organ system dysfunctions related to the disease burden of postoperative sepsis episodes are unclear.

**Methods:**

We analyzed epidemiological characteristics of postoperative sepsis based on the ICD-9-CM codes for the years 2002 to 2013 using the Longitudinal Health Insurance Databases of Taiwan’s National Health Insurance Research Database.

**Results:**

We identified 5,221 patients with postoperative sepsis and 338,279 patients without postoperative sepsis. The incidence of postoperative sepsis increased annually with a crude mean of 0.06% for patients aged 45–64 and 0.34% over 65 years. Patients with postoperative sepsis indicated a high risk associated with the characteristics, male sex (OR:1.375), aged 45–64 or ≥ 65 years (OR:2.639 and 5.862), low income (OR:1.390), aged township (OR:1.269), agricultural town (OR:1.266), and remote township (OR:1.205). Splenic surgery (OR:7.723), Chronic renal disease (OR:1.733), cardiovascular dysfunction (OR:2.441), and organ system dysfunctions had the highest risk of postoperative sepsis.

**Conclusion:**

Risk of postoperative sepsis was highest among men, older, and low income. Patients with splenic surgery, chronic renal comorbidity, and cardiovascular system dysfunction exhibited the highest risk for postoperative sepsis. The evaluation of high-risk factors assists in reducing the disease burden.

## 1 Introduction

Sepsis is the most severe manifestation of acute infection, causing a complex syndrome that may result in multiple organ failures and resulting in death in 30–50% cases [1, 2]. Sepsis, a major cause of morbidity and mortality worldwide, is one of the 10 leading causes of death in Taiwan and the United States [3]. The incidence of sepsis is increasing. In the United States, approximately 164,000 cases of sepsis occurred each year during the 1970s [4]. However, according to studies performed by the National Center for Health Statistics, the incidence of sepsis has risen from 221 cases per 100,000 persons in 2000 to 377 per 100,000 persons in 2008, which is an increase of 7%–8% per year [5, 6]. In Taiwan, a nationwide population-based study demonstrated that the incidence of severe sepsis rose from 135 cases per 100,000 persons in 1997 to 217 per 100,000 persons in 2006, which is an approximately 3.9% increase per year [7]. The medical cost for treating sepsis has increased along with necessity for medical equipment, medical supplies, and healthcare services associated with sepsis improvement [8].

Postoperative sepsis is defined by the Agency for Healthcare Research and Quality according to the Ninth Revision and Clinical Modification codes of the International Classification of Diseases (ICD-9-CM) [9]. It includes patients 18 years or older who have undergone surgical procedures and have had postoperative hospital stays for no fewer than 4 days [9]. The major form of sepsis is postoperative sepsis, which accounts for approximately one-third of all sepsis cases [3]. Postoperative sepsis results in significant morbidity and mortality and is a leading cause of multiple organ dysfunction and death for hospital inpatients [10, 11]. However, the understanding of the incidence and temporal trends of postoperative sepsis in Taiwan has remained limited because only a few studies have been proposed to investigate these trends. The incidence and temporal trends of postoperative sepsis are critical information for developing strategies for treatment and reducing the cost of intervention.

The primary goal of this study was to discern the incidence and temporal trends of postoperative sepsis in Taiwan using a retrospective population-based survey. The secondary objective was to compare the incidence of postoperative sepsis after surgery with respect to various types of procedures. Moreover, we evaluated sociodemographic characteristics, comorbidities, and organ system dysfunctions associated with the development of postoperative sepsis. The present study identified the high-risk population from the database because doing so might assist with identifying process-level opportunities for reducing the incidence of postoperative sepsis.

## 2 Materials and methods

### 2.1 Data source

The data used in this study were collected from the Longitudinal Health Insurance Databases (LHID 2010) of the National Health Insurance Research Database (NHIRD) for the years 2002 to 2013. The NHIRD was released for research purposes by the Taiwan National Health Research Institutes and covers nearly all patient medical benefit claims for the Taiwanese population. The NHIRD, which is one of the largest and most detailed nationwide population-based datasets in the world, is widely used in epidemiological research.

### 2.2 Study population selection and definitions

**Figure 1:**
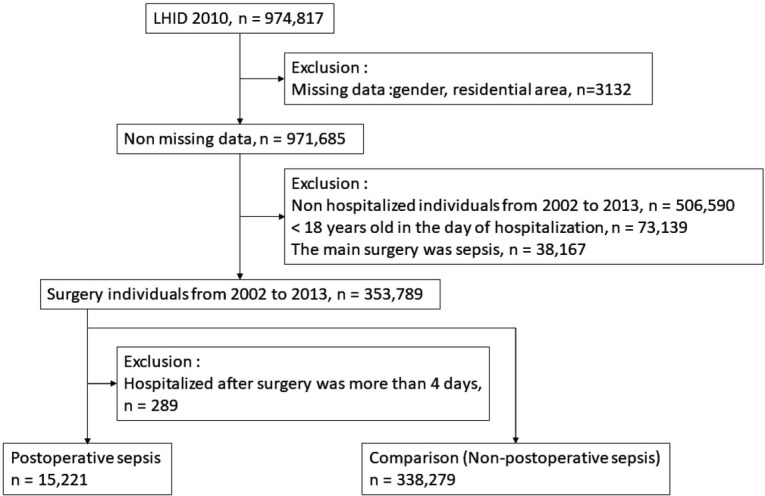
Case selection procedure

This study population comprised data regarding 974,817 patients collected from the LHID 2010 (Figure 1). Missing values for variables such as sex and residential area were excluded. Individuals who were not hospitalized, under 18 years of age, or had sepsis before surgery were also excluded. Using ICD-9-CM codes, we identified patients according to category of surgical procedure, and both hospitalized patients and surgical diagnosis-related groups (DRGs) were selected [9, 11, 12, 13]. The sepsis population was identified by secondary diagnoses using ICD-9-CM codes, namely 038, 038.0, 038.1, 038.2, 038.3, 038.4, 038.8, 038.9, 038.10, 038.11, 038.19, 038.40, 038.41, 038.42, 038.43, 038.44, 038.49, 790.7, and 996.62. In addition, all patients selected for the study had stayed in the hospital for no fewer than 4 days.

Twelve major groups of surgical procedures, according to the ICD-9-CM codes for principal procedures, were included in the data analysis, namely esophageal, pancreatic, small bowel, gastric, splenic, gallbladder, hepatic, vascular, colorectal, thoracic, cardiac, and hernia surgeries. Sociodemographic characteristics included sex, age (divided into the following age groups: 20–40, 40–65, and ≥ 65 years), income level, and urbanization level [12]. Comorbidities included carcinoma in situ (ICD-9-CM: 230–234), diabetes (ICD-9-CM: 272), chronic heart failure (ICD-9-CM: 428), unspecified hepatitis (ICD-9-CM: 070.9, 571.4, 571.8, 571.9), chronic obstructive pulmonary disease (COPD, ICD-9-CM: 490-505 506.4), chronic renal disease (ICD-9-CM: 585), and acute myocardial infarction (ICD-9-CM: 410). Organ system dysfunctions included acute renal disease (ICD-9-CM: 584, 580, 39.95) and dysfunction of the respiratory (ICD-9-CM: 518.81–518.85, 786.09, 799.1, 96.7), cardiovascular (ICD-9-CM: 785.5, 458, 796.3), hepatic (ICD-9-CM: 570, 572.2, 573.3), hematological (ICD-9-CM: 286.6, 286.9, 287.3-287.5), and metabolic (ICD-9-CM: 276.2) systems [11,13].

### 2.3 Statistical analysis

Incidence was estimated using the total number of LHID 2010 cases with a surgical DRG code involving patients aged 18 years or older. Age-adjusted rates were calculated using direct methods with respect to the surgical in-hospital population from 2002 to 2013. The chi-squared test was used to assess the nominal variables between patients with and without postoperative sepsis. Logistic regression was used to estimate the odds ratio (OR) and 95% confidence interval (CI) for patients with and without postoperative sepsis. Data analysis was performed using SAS 9.3 software (SAS Institute, Cary, NC), and a *P* value < 0.05 was considered statistically significant.

## 3 Results

**Figure 2:**
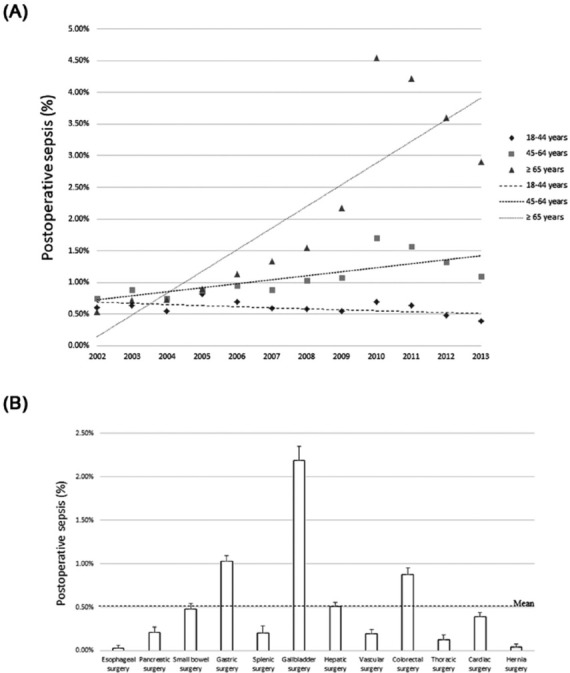
(A) Incidence of postoperative sepsis by age group (B) Incidence of postoperative sepsis by surgery type

The final enrollment included 15,221 patients with postoperative sepsis and 338,279 patients without postoperative sepsis during the study period (Figure 1). The incidence of postoperative sepsis increased annually with a crude mean of 0.06% and 0.34% for patients aged 45–64 and ≥ 65 years, respectively (Figure 2A), and the incidence declined slightly with a crude mean of 0.01% for patients aged 18–44 years (Figure 2A). Figure 2B presents the average annual incidence of sepsis after various surgical procedures. Gastric, gallbladder, and colorectal surgeries were associated with a relatively high likelihood of postoperative sepsis, and esophageal surgery had the lowest risk of postoperative sepsis.

The distribution of sociodemographic characteristics, comorbidities, and organ system dysfunctions among the study population are presented in Table 1. The differences between patients with postoperative sepsis and those without were significant with respect to sociodemographic characteristics, namely sex, age, income level, and urbanization level (*P* < 0.001). These differences in patients who had undergone various surgical procedures were also significant (*P* < 0.001), except for gallbladder surgery (*P* = 0.6620). In addition, the differences in patients with comorbidities were significant (*P* < 0.001), except for the comorbidity of carcinoma in situ (*P* = 0.1886).

**Table 1:** Basic characteristics of the study participants

	Postoperative non-sepsis (n = 338279)		Postoperative sepsis (n = 15221)		P-value
**Gender**					<.0001
female	189467	(56.01%)	6834	(44.90%)	
male	148812	(43.99%)	8387	(55.10%)	
**Age**					<.0001
18-44 years	222761	(65.85%)	3752	(24.65%)	
45-64 years	47336	(13.99%)	2389	(15.70%)	
≥65 years	68182	(20.16%)	9080	(59.65%)	
**Low income**					<.0001
Yes	170559	(50.42%)	3752	(37.07%)	
No	167720	(49.58%)	2389	(62.93%)	
**Urbanization level**					<.0001
Highly urbanized	98837	(29.22%)	3851	(25.30%)	
Moderate urbanization	100507	(29.71%)	4331	(28.45%)	
Emerging town	59945	(17.72%)	2376	(15.61%)	
General town	47102	(13.92%)	2429	(15.96%)	
Aged Township	6507	(1.92%)	504	(3.31%)	
Agricultural town	12855	(3.8%)	936	(6.15%)	
Remote township	12526	(3.7%)	794	(5.22%)	
**Surgical procedures**					
Esophageal surgery	65	(0.02%)	8	(0.05%)	<.0001
Pancreatic surgery	98	(0.03%)	22	(0.14%)	<.0001
Small bowel surgery	261	(0.08%)	67	(0.44%)	<.0001
Gastric surgery	911	(0.27%)	141	(0.93%)	<.0001
Splenic surgery	95	(0.03%)	20	(0.13%)	<.0001
Gallbladder surgery	4789	(1.42%)	222	(1.46%)	0.6620
Hepatic surgery	809	(0.24%)	60	(0.39%)	0.0002
Vascular surgery	200	(0.06%)	30	(0.20%)	<.0001
Colorectal surgery	1504	(0.44%)	119	(0.78%)	<.0001
Thoracic surgery	1043	(0.31%)	19	(0.12%)	<.0001
Cardiac surgery	762	(0.23%)	51	(0.34%)	<.0001
Hernia surgery	6432	(1.90%)	12	(0.08%)	<.0001
**Comorbidities**					
Carcinoma in situ	2988	(0.88%)	150	(0.99%)	0.1886
Diabetes	59130	(17.48%)	6514	(42.80%)	<.0001
Chronic heart failure	22580	(6.67%)	3824	(25.12%)	<.0001
COPD	96522	(28.53%)	7760	(50.98%)	<.0001
Chronic renal disease	11755	(3.47%)	2451	(16.1%)	<.0001
Chronic liver disease	280	(0.08%)	28	(0.18%)	<.0001
Acute myocardial infarction	3386	(1.00%)	483	(3.17%)	<.0001
**Organ system dysfunction**					
Respiratory	16554	(4.89%)	2573	(16.9%)	<.0001
Cardiovascular	5273	(1.56%)	1192	(7.83%)	<.0001
Acute renal disease	2522	(0.75%)	532	(3.50%)	<.0001
Hepatic	16086	(4.76%)	1282	(8.42%)	<.0001
Haematological	2439	(0.72%)	351	(2.31%)	<.0001
Neurological	380	(0.11%)	63	(0.41%)	<.0001
Metabolic	278	(0.08%)	75	(0.49%)	<.0001

Included postoperative sepsis and comparison: non postoperative sepsis.

Table 2 presents the logistic regression analysis of patients with and without postoperative sepsis. Adjustments were made for sex, age, income level, urbanization level, surgical procedures, comorbidities, and organ system dysfunctions. Among the sociodemographic characteristics, the results indicated a high risk of postoperative sepsis among the following categories of patients: men (OR 1.375, 95% CI 1.375–1.423), aged 45–64 and ≥ 65 years (OR 2.639 and 5.862, 95% CI 2.496–2.790 and 5.545–6.196), low income (OR 1.390, 95% CI 1.134–1.441), aged township (OR 1.269, 95% CI 1.147–1.403), agricultural town (OR 1.266, 95% CI 1.172–1.368), and remote township (OR 1.205, 95% CI 1.110–1.308). The aged township category was associated with the highest risk with respect to urbanization level for postoperative sepsis among patients. Among patients who underwent other surgical procedures, those who underwent esophageal, pancreatic, small bowel, gastric, splenic, and vascular surgeries had the highest risk of postoperative sepsis. Among these procedures, splenic surgery (OR 7.723, 95% CI 4.614–12.927) had the highest risk. The specific comorbidities and organ system dysfunctions associated with the highest risk of postoperative sepsis were diabetes, chronic heart failure, COPD, chronic renal disease, respiratory dysfunction, cardiovascular dysfunction, acute renal disease, hepatic dysfunction, hematological dysfunction, neurological dysfunction, and metabolic dysfunction. Patients with chronic renal disease (OR 1.733, 95% CI 1.644–1.827) and cardiovascular dysfunction (OR 2.441, 95% CI 2.272–2.622) had the highest risks among comorbidities and organ system dysfunctions, respectively. These results indicated that patients who were men, older adults, lived in an aged township, had undergone splenic surgery, or had received a diagnosis of chronic renal disease or cardiovascular dysfunction had a relatively high risk of postoperative sepsis.

**Table 2:** Odds ratio and 95% confidence interval of surgery among patients with sepsis

	Odds ratio (95%CI)	P-value
**Gender (reference: female)**		
Male	1.375 (1.328-1.423)	<.0001
**Age (reference: 18-44 years old)**		
40-64 years	2.639 (2.496-2.790)	<.0001
≥ 65 years	5.862 (5.545-6.196)	<.0001
**Low income (reference : no)**		
Yes	1.390 (1.34-1.441)	<.0001
**Urbanization level (reference:Moderate urbanization)**		
Highly urbanized	0.937 (0.895-0.981)	0.0056
Emerging town	0.938 (0.890-0.989)	0.0181
General town	1.037 (0.983-1.094)	0.1876
Aged township	1.269 (1.147-1.403)	<.0001
Agricultural town	1.266 (1.172-1.368)	<.0001
Remote township	1.205 (1.110-1.308)	<.0001
**Surgical procedures (reference: other surgery)**		
Esophageal surgery	2.668 (1.217-5.847)	0.0143
Pancreatic surgery	4.045 (2.466-6.635)	<.0001
Small bowel surgery	4.242 (3.170-5.675)	<.0001
Gastric surgery	2.927 (2.420-3.539)	<.0001
Splenic surgery	7.723 (4.614-12.927)	<.0001
Gallbladder surgery	1.032 (0.898-1.186)	0.6583
Hepatic surgery	1.168 (0.890-1.534)	0.2636
Vascular surgery	1.962 (1.305-2.949)	0.0012
Colorectal surgery	1.184 (0.976-1.437)	0.0862
Thoracic surgery	0.431 (0.272-0.683)	0.0003
Cardiac surgery	0.763 (0.569-1.022)	0.0696
Hernia surgery	0.034 (0.019-0.060)	<.0001
**Comorbidities (reference: without)**		
Carcinoma in situ	0.830 (0.699-0.985)	0.0332
Diabetes	1.494 (1.439-1.550)	<.0001
Chronic heart failure	1.414 (1.351-1.480)	<.0001
COPD	1.149 (1.107-1.192)	<.0001
Chronic renal disease	1.733 (1.644-1.827)	<.0001
Chronic live disease	0.568 (0.373-0.863)	0.0081
Acute myocardial infarction	1.045 (0.939-1.163)	0.4193
**Organ system dysfunction (reference: without)**		
Respiratory	1.785 (1.697-1.877)	<.0001
Cardiovascular	2.441 (2.272-2.622)	<.0001
Acute renal disease	1.573 (1.416-1.747)	<.0001
Hepatic	1.194 (1.121-1.271)	<.0001
Haematological	1.699 (1.504-1.919)	<.0001
Neurological	1.140 (0.858-1.514)	0.3665
Metabolic	1.667 (1.259-2.206)	0.3665 0.0004

Abbreviation: CI, confidence interval, COPD, chronic obstructive pulmonary disease.

Table 3 presents the sociodemographic characteristics and risk of postoperative sepsis in each of the predefined age groups, which were 18–44, 45–64, and ≥65 years old. In the 18–44 age group, the following characteristics of patients were associated with a relatively high risk of postoperative sepsis: male sex, low income, remote township, pancreatic surgery, diabetes, and cardiovascular dysfunction. In the 45–64 age group, the high-risk characteristics of patients associated with postoperative sepsis were male sex, low income, aged township, small bowel surgery, chronic renal disease, and cardiovascular dysfunction. In the ≥65 age group, the high-risk characteristics postoperative sepsis were male sex, low income, agricultural town, splenic surgery, chronic renal disease, and cardiovascular dysfunction.

**Table 3:** Odds ratio and 95% confidence interval of surgery among patients with sepsis in each of the predefined age groups

	Odds ratio (95%CI) 18-44 years (n=2008)		45-64 years (n=4133)		≥ 65 years (n=9080)	
**Gender (reference: female)**						
Male	1.469 (1.343-1.608)	***	1.585 (1.484-1.692)	***	1.234 (1.179-1.291)	***
**Low income (reference : no)**						
Yes	1.219 (1.114-1.333)	***	1.635 (1.534-1.743)	***	1.273 (1.206-1.344)	***
**Urbanization level (reference: Moderate urbanization)**						
Highly urbanized	0.952 (0.847-1.069)		0.937 (0.862-1.019)		0.940 (0.883-1.001)	
Emerging town	0.923 (0.808-1.053)		0.958 (0.869-1.056)		0.920 (0.856-0.989)	*
General town	0.963 (0.830-1.118)		0.983 (0.886-1.090)		1.032 (0.961-1.108)	
Aged Township	1.016 (0.701-1.472)		1.636 (1.336-2.002)	***	1.126 (0.994-1.275)	
Agricultural town	1.406 (1.120-1.765)	**	1.352 (1.161-1.574)	**	1.141 (1.033-1.260)	**
Remote township	1.466 (1.181-1.820)	**	1.365 (1.168-1.596)	***	1.041 (0.934-1.160)	
**Surgical procedures (reference: other surgery)**						
Esophageal surgery	2.588 (0.338-19.815)		3.166 (1.112-9.017)	*	2.094 (0.530-8.283)	
Pancreatic surgery	11.497 (3.870-34.159)	***	4.900 (2.313-10.378)	***	2.528 (1.181-5.410)	*
Small bowel surgery	9.715 (4.873-19.365)	***	6.701 (4.127-10.880)	***	2.673 (1.796-3.978)	***
Gastric surgery	2.569 (1.460-4.521)	**	2.424 (1.695-3.466)	***	3.141 (2.451-4.025)	***
Splenic surgery	9.534 (4.646-19.565)	***	6.371 (2.726-14.890)	***	4.042 (1.030-15.863)	*
Gallbladder surgery	0.393 (0.195-0.789)	**	0.957 (0.758-1.208)		1.223 (1.019-1.467)	*
Hepatic surgery	1.710 (0.665-4.397)		1.643 (1.102-2.450)	*	0.861 (0.579-1.282)	
Vascular surgery	7.714 (2.341-25.418)	**	2.801 (1.442-5.439)	**	1.374 (0.793-2.381)	
Colorectal surgery	4.876 (2.687-8.850)	***	1.326 (0.934-1.883)		0.967 (0.756-1.238)	
Thoracic surgery	0.596 (0.222-1.601)		0.408 (0.168-0.990)	*	0.402 (0.212-0.761)	**
Cardiac surgery	5.701 (2.897-11.22)	***	0.515 (0.273-0.973)	*	0.661 (0.454-0.962)	*
Hernia surgery	0.042 (0.006-0.298)	**	0.030 (0.010-0.093)	***	0.035 (0.017-0.070)	***
**Comorbidities (reference: without)**						
Carcinoma in situ	0.865 (0.428-1.747)		0.789 (0.557-1.119)		0.867 (0.706-1.064)	
Diabetes	2.780 (2.432-3.179)	***	1.743 (1.628-1.865)	***	1.274 (1.217-1.333)	***
Chronic heart failure	1.903 (1.416-2.557)	***	1.465 (1.319-1.626)	***	1.377 (1.309-1.448)	***
COPD	1.021 (0.912-1.143)		1.041 (0.972-1.115)		1.231 (1.173-1.292)	***
Chronic renal disease	2.682 (2.015-3.569)	***	2.456 (2.206-2.735)	***	1.559 (1.467-1.657)	***
Chronic liver disease	1.187 (0.104-13.506)		0.649 (0.250-1.679)		0.546 (0.341-0.873)	*
Acute myocardial infarction	1.422 (0.645-3.133)		0.924 (0.729-1.172)		1.091 (0.968-1.231)	
		
**Organ system dysfunction (reference: without)**						
Respiratory	1.714 (1.404-2.093)	***	1.732 (1.554-1.931)	***	1.783 (1.681-1.892)	***
Cardiovascular	4.668 (3.739-5.827)	***	2.998 (2.590-3.470)	***	2.116 (1.942-2.305)	***
Acute renal disease	2.271 (1.490-3.460)	**	1.723 (1.388-2.139)	***	1.458 (1.289-1.648)	***
Hepatic	1.723 (1.440-2.061)	***	1.358 (1.222-1.510)	***	0.990 (0.908-1.079)	
Haematological	3.953 (2.808-5.565)	***	2.654 (2.141-3.290)	***	1.242 (1.061-1.453)	**
Neurological	3.037 (1.057-8.729)	*	2.275 (1.259-4.112)	**	0.936 (0.672-1.304)	
Metabolic	2.178 (0.817-5.804)		1.425 (0.838-2.425)		1.603 (1.137-2.261)	**

Abbreviation: CI, confidence interval, COPD, chronic obstructive pulmonary disease. * *, **, *** Statistically significant, respectively P <0.05, P <0.01, P <0.0001.

## 4 Discussion

Taiwan launched the National Health Insurance (NHI) program in March 1995 to provide single-payer health insurance to residents of Taiwan; now, the NHI covers more than 99% of the population and enables them to easily access affordable healthcare with only a small copayment required at most clinics and hospitals. To the best of our knowledge, a retrospective and longitudinal nationwide study of postoperative sepsis has not been undertaken in Taiwan. However, nationwide databases have been used widely in the United States to identify the changes over time concerning the incidence, epidemiological characteristics, and temporal trends in postoperative sepsis in the country [11, 15]. The incidence of postoperative sepsis in the United States increased from 4.41% in 1990 to 6.25% in 2006 according to the Nationwide Inpatient Sample dataset [14]. The same study also demonstrated that the incidence of postoperative sepsis was 3.4% among those aged 18–49 years but 6.9% among those aged ≥80 years [14]. No evidence has been compiled and analyzed to assess the incidence of postoperative sepsis in Taiwan. However, one study analyzed data gathered from the NHIRD and determined the incidence of sepsis had increased from 637.8 per 100,000 persons in 2002 to 772.1 per 100,000 persons in 2012 [16]; the study also discovered that the incidence of sepsis in older adults (aged ≥65 years) was 10-fold higher than in younger adults (18–64 years) of the same sex [16]. In the present study, we determined that the incidence of postoperative sepsis increased from 1.90% in 2002 to 4.40% in 2013. Older adults (≥65 years) had a greater increase in incidence of postoperative sepsis than did middle-aged patients (45–64 years); however, the incidence of postoperative sepsis in younger (18–44 years) patients decreased slightly over the same period. This finding may imply that the senescence of older adult patients’ organs results in their higher incidence of postoperative sepsis.

The average annual incidence of sepsis with respect to a variety of surgical procedures is presented in Figure 2. We discovered higher incidences of sepsis associated with gastric, gallbladder, and colorectal surgeries; however, no statistically significant difference was evident with respect to the incidence of sepsis after undergoing gallbladder surgery. These results indicated that the number of patients who underwent gallbladder surgery was the largest among all the surgical procedures and thus led to the highest average annual incidence of sepsis after surgery. These findings were similar to those regarding the average annual incidence of sepsis after colorectal surgeries. Moreover, we determined that patients had a higher likelihood of developing sepsis after undergoing general abdominal surgeries such as splenic, small bowel, pancreatic, gastric, and esophageal surgeries. The basic rule in abdominal surgeries is to have an incision that offers the appropriate passageway to the region in the abdominal organ or tissue which suffered damage through injury or disease [17]. Pathologists have hypothesised that patients have higher risk of sepsis via microbial infection after spleens are processed as surgical specimens, splenic implants, or spleen removal surgery [18].

After performing the surgical removal of all or part of the pancreas, an increased the risk for the development of sepsis was found [19]. After radical gastrectomy for gastric cancer, post-operative infection is the most common complication via leukocyte depletion in transfusion patient [20]. Postoperative infection is the lethal and hospitalized factor in patients who underwent lower gastrointestinal surgery of the small intestine, colon, rectum, or anus in Unit State [21]. Studies had shown that patients who underwent surgical esophagectomy had a high risk of postoperative complications such as sepsis and high mortality [22]. These findings were similar to those discovered by researchers in the United States, indicating that the highest risks of postoperative sepsis with respect to general abdominal procedures were associated with esophageal, gastric, pancreatic, small bowel, and biliary surgeries [11]. And intra-abdominal infection is a common cause of open laparotomy in non-traumatic patients [23]. This study identified that pancreatic, small bowel, and splenic surgeries were associated with higher risks among patients aged 18–44, 45–64, and ≥65 years, respectively. In addition, there is much evidence claiming that postoperative sepsis was found in patients after an uncomplicated colonoscopic polypectomy, which is regarded as a safe procedure, due to unidentified abdominal infections [24, 25]. These results suggest that general abdominal procedures, including not only complex surgeries but also routine procedures, pose a significant risk for the development of postoperative sepsis.

Our logistic regression analysis demonstrated that several sociodemographic characteristics of the patients influenced their risk of postoperative sepsis. Being male or older (≥65 years) considerably increased the risk of developing postoperative sepsis; the elevated risk associated with these factors has been consistently reported in other epidemiological studies of sepsis [16, 26, 27] and postoperative sepsis [14, 15]. Sex hormones play a major role in shaping the host’s response to sepsis [28, 29, 30]. Older patients may have an increased risk of postoperative sepsis because of declining immune system performance and age-associated immunosenescence [31, 32]. Thus, our evidence lends support to the contention that male sex and older age may play key roles in the development of postoperative sepsis because of the regulation of the expression of sex hormones and defective immunological function, respectively.

Several possible comorbidities and organ system dysfunctions were associated with an increased incidence of postoperative sepsis. The comorbidities of chronic renal disease, diabetes, and chronic heart failure were associated with the first, second, and third highest risks, respectively, of patients developing postoperative sepsis. In addition, chronic renal disease, diabetes, and chronic heart failure were associated with a higher likelihood of developing postoperative sepsis among all age groups. Similar to the findings of other studies, the aforementioned chronic comorbidities increased the likelihood of sepsis [33, 34, 35]. A possible reason for patients with these three chronic comorbidities to have a higher risk of developing postoperative sepsis could be because they are each related to an inflammatory response that results in fewer and worse functioning leukocytes. Such evidence suggests that these three chronic comorbidities might play major roles in the development of postoperative sepsis attributable to the generation of inflammation and immune system dysfunction.

Organ system dysfunction was analyzed for all patients and age groups, and cardiovascular and hematological system dysfunctions were associated with the highest likelihood of developing postoperative sepsis. Organ system dysfunction was also associated with increased risk of sepsis [13]. These findings supported the findings of other studies that cardiovascular and hematological systems play major roles in the development of postoperative sepsis.

## 5 Limitations of the study

The present study has several limitations. The LHID 2010 and NHIRD are derived from a large administrative database. Although many studies have validated the use of this administrative data [11, 13], the data were originally intended for claims reimbursements and thus lack detailed biochemical data for each individual. Moreover, a statistical bias attributable to the coding schemes related to the limitations of various clinical entities cannot be entirely excluded; for example, they may have failed to accurately differentiate comorbidities from chronic diseases and organ system dysfunctions [11, 13, 15]. Some comorbidities or organ system dysfunctions may have been missed because only five diagnostic ICD-9-CM codes were assessed, which may have resulted in an underestimation of the incidence. An intrinsic bias may be attributable to the use of the ICD-9-CM discharge code, which may have been applied differently between and even within institutions.

## 6 Conclusions

The factors related to increased incidences of postoperative sepsis in our study were similar to those in studies undertaken in the United States. After general abdominal surgeries, namely splenic, small bowel, pancreatic, gastric, and esophageal surgeries, patients had a higher likelihood of developing sepsis. Patients who were male and older exhibited a considerably higher risk of developing postoperative sepsis than patients in other categories. Chronic renal disease, diabetes, chronic heart failure, and organ system dysfunction, including cardiovascular and hematological system dysfunction, were associated with a higher risk of developing postoperative sepsis in both the differential age groups and all patients. The key finding of this study was the valuable predictors associated with an increased risk of developing postoperative sepsis; these predictors, namely being male or older, undergoing splenic surgery, and having chronic renal comorbidities or cardiovascular system dysfunction, present a potential target for future research to reduce the disease burden.
